# Characterization of the rat pituitary capsule: Evidence that the cerebrospinal fluid filled the pituitary cleft and the inner side of the capsule

**DOI:** 10.1371/journal.pone.0286399

**Published:** 2023-05-26

**Authors:** Edgar Giovanhi Gómez-Domínguez, César Gabriel Toriz, Sirenia González-Pozos, Margarita González-del-Pliego, Elsa Liliana Aguirre-Benítez, Armando Pérez-Torres, Yazmin Monserrat Flores-Martinez, Carmen Solano-Agama, Verónica Rodríguez-Mata, Alejandro García-Godínez, Daniel Martínez-Fong, María Eugenia Mendoza-Garrido

**Affiliations:** 1 Departamento de Fisiología, Biofísica y Neurociencias, Centro de Investigación y de Estudios Avanzados, Instituto Politécnico Nacional, Ciudad de México, México; 2 Coordinación General de Servicios Experimentales, Microscopía Electrónica, Centro de Investigación y de Estudios Avanzados, Instituto Politécnico Nacional, Ciudad de México, México; 3 Departamento de Embriología, Facultad de Medicina, Universidad Nacional Autónoma de México, Ciudad de México, México; 4 Departamento de Biología Celular y Tisular, Facultad de Medicina, Universidad Nacional Autónoma de México, Ciudad de México, México; Federal University of Rio de Janeiro, BRAZIL

## Abstract

In humans, the pituitary gland is covered by a fibrous capsule and is considered a continuation of the meningeal sheath. However, in rodents some studies concluded that only the *pars tuberalis* (PT) and *pars nervosa* (PN) are enwrapped by the pia mater, while others showed that the whole gland is covered by this sheath. At PT the median eminence subarachnoid drains cerebrospinal fluid (CSF) to its cisternal system representing a pathway to the hypothalamus. In the present study we examined the rat pituitary capsule to elucidate its configuration, its physical interaction with the pituitary border and its relationship with the CSF. Furthermore, we also revisited the histology of the pituitary cleft and looked whether CSF drained in it. To answer such questions, we used scanning and transmission electron microscopy, intracerebroventricular infusion of Evan´s blue, fluorescent beads, and sodium fluorescein. The latter was measured in the *pars distalis* (PD) and various intracranial tissues. We found a pituitary capsule resembling leptomeninges, thick at the dorsal side of the *pars intermedia* (PI) and PD, thicker at the level of PI in contiguity with the PN and thinner at the rostro-ventral side as a thin membrane of fibroblast-like cells embedded in a fibrous layer. The capsule has abundant capillaries on all sides. Our results showed that the CSFs bathe between the capsule and the surface of the whole gland, and ciliate cells are present in the pituitary border. Our data suggest that the pituitary gland intercommunicates with the central nervous system (CNS) through the CSF.

## Introduction

The pituitary gland is an intracranial organ, localized below the hypothalamus and lying on the sphenoid bone, and originates from the invagination of the Rathke´s pouch and an evagination of the infundibulum. The rodent pituitary gland is constituted of a posterior hypophysis or *pars nervosa* (PN), and an anterior hypophysis or adenohypophysis composed of *pars intermedia* (PI), *pars distalis* (PD) and *pars tuberalis* (PT). Being an intracranial gland and bathed by the cerebrospinal fluid (CSF), the pituitary gland is encapsulated by the dura mater, while the basal hypothalamus, including the median eminence, the PN, and pituitary stalk or PT, are covered by the pia mater and dura mater [1]. However, a fibrous capsule with variable thickness surrounding the anterior hypophysis is observed in human pituitary [2, 3]. According to Krisch and Buchheim [4], and in disagreement with Mezey and Palkovits [1], a meningeal thin sheath envelops the gland in rats. The function of the anterior pituitary or adenohypophysis is regulated by hypothalamic neurohormones secreted at the median eminence, which is part of the circumventricular organs [5, 6]. Even though the portal vessels are constituted of fenestrated capillaries, in the media eminence, separation between the cerebral milieu and the peripheral blood milieu is maintained. However, in the vicinity of the median eminence, the PT is exposed to the CSF from the subarachnoid space [7, 8] which is a route to the hypothalamus of the messengers generated by the PT transduction of the photoperiodic signals [9]. CSF has been observed to bathe the PT through a system based on a cistern structure where the bordering cells present abundant 9+2 cilia projecting to the lumen, and other cells exhibit a unique cilia and microvilli, resembling the typical follicle-stellate cells present in the PD [8]. In addition, the PT tissue is composed of endocrine cells that exhibit some peculiarities, differentiating them from the PD endocrine cells, as single 9+0 cilia that project to the intercellular channels and are the secretory cell population of the midline of this pituitary region [8]. Interestingly, at the epithelial luminal border of the pituitary cleft, Ciocca [10] and Correr and Mota [11] observed multiciliate cells and cells with microvilli at the cellular borders in the posterior epithelial surface. At the anterior epithelial border there are fewer cells with these characteristics. Correr and Mota [11] also observed cells loosely attached at the anterior epithelial surface and they postulated that the lacunar spaces present at PD tissue could drain to the cleft. Then, PT is bathed by the CSF and is part of Rathke´s pouch development, and the pituitary cleft presents cells very like those from PT, suggesting that CSF could drain to Rathke´s cleft in the rat pituitary. We analysed this possibility by injecting a dye into a lateral ventricle and looking for its presence at the pituitary cleft. Moreover, we identified that the pituitary capsule resembling the leptomeninges covering PI and the posterior surface of the PD becomes thinner as it runs along the ventral surface. This capsule is rich in continuous non-fenestrated capillaries. The CSF fills the space between the basal domain of the pituitary cells, which is closely opposed to the basal lamina, and the capsule. We proposed that not only PT is in contact with the CSF since the pituitary cleft is also filled with CSF. Moreover, the superficial cells of the PD can receive signals from the CSF, as suggested by the presence of cilia in these cells. Our data suggest that the pituitary gland intercommunicates with the CNS through the CSF using the volume transmission communication system [12, 13].

## Materials and methods

### Animals

Adult (60–90 days old) “Wistar” rats grew in our colony and maintained with constant room temperature (21 ± 1°C) and humidity (55%) with a dark/light cycle of 12 h beginning the dark period at 18:00 h, with food and water provided *ad libitum*. All the procedures described in this report were approved by the Cinvestav Animal Used and Care Ethical Committee (CICUAL, # 0267–05), following the Mexican Official Rule NOM-062-ZOO-1999 and the Guide from the National Institutes of Health (NIH-USA, #8023). There were 35 rats used in the present study.

### Pituitary glands

The pituitary glands were obtained from perfused animals or from decapitated animals. The perfusion was developed in rats anaesthetized with 60 mg/kg body weight sodium pentobarbital (Pentobarbital®, Aranda, MX) and perfused through the left heart ventricle with 2.5% glutaraldehyde in 0.15 M sodium cacodylate buffer (pH 7.4) or 4% paraformaldehyde plus 3% sucrose in phosphate-buffer saline (PBS) 100 ml (pH 7.4). The large vessels, both vena cava and aorta descendent, were clamped prior to perfusion. In some experiments, PBS was perfused first followed by the fixer solution. The rate of perfusion was 1 drop/sec of 100 ml. After fixation, the pituitary gland was dissected, immersed in fixer solution, and processed for microscopic observation. Some glands were obtained from decapitated rats and the glands were fixed by immersion with 2.5% glutaraldehyde in 0.15 M sodium cacodylate buffer (pH 7.4) for 4 h at 4°C.

### Scanning Electron Microscopy

Pituitary glands obtained after the rats were perfused with glutaraldehyde were separated into two groups: a group of glands transversally cut and the other used the intact gland, the latter to observe the whole surface of the gland. Both groups of glands were immersed in the same fixative plus sucrose 2% at 4°C overnight, followed by a treatment with 1% tannic acid in the same fixative solution at room temperature for 2 h in darkness. Then, the glands were rinsed and postfixed with 2% OsO_4_ plus 2% sucrose in cacodylate buffer for 2 h followed by an incubation with 1% OsO_4_ overnight at 4°C. After several washes the glands or the cuts were dehydrated with ethanol (5 to 100%) and subsequently infiltrated with isoamyl acetate (Sigma, Burlington, MA, USA) and dried with hexamethyldisilazane (HMDS, Electron Microscopy Sciences, Philadelphia, PA, USA) in a desiccator for 48 h. The samples were gold-sputtered and analysed under a scanning electron microscope (JSM-6510LV, JEOL, Tokyo, Japan). In some experiments were used the Tanaka´s technique, using a double-sided adhesive carbon tape the tissue is attached to it and then its pull. This technique was used to observe what was under the fibrous layer covering the PD.

### Transmission Electron Microscopy

Pituitary glands obtained from decapitated rats were cut into small pieces of approximately 1–2 mm, placed in glutaraldehyde solution and postfixed with 1% OsO_4_ in cacodylate buffer 0.15 M 2 h, at room temperature. Then, the tissues were washed and dehydrated in a graded series of ethanol from 30% to 100%, followed by propylene oxide and embedded in Embed 812 (Electron Microscopy Sciences). Ultrathin sections (60–90 nm) were mounted on nickel grids (Polysciences Inc, Philadelphia, PA, USA). The sections were rehydrated in distilled water drops for 10 min and counterstained with 4% uranyl acetate for 20 min, followed by 2.5% lead citrate for 7 min, washed several times with distilled water and dried. Sections were examined, and electron micrographs were taken by using an electron microscope (Crossbeam 550, Gemini 2, Zeiss, Jena, Germany). Semithin sections (0.5 μm) were also obtained, stained with toluidine blue and mounted in Entellan (Merck Millipore, Darmstadt, Germany).

### Intracerebral ventricular injection

Anaesthetized rats were held in a stereotaxic frame (Stoelting Co, Chicago, IL, USA) to trepan their skulls with a dental drill at the appropriate location. A 0.5% Evan´s blue solution in PBS (Sigma-Aldrich, St. Louis, MO, USA) or 6 mg/ml sodium fluorescein (NaFluo) in PBS was injected into the left lateral ventricle at the following coordinates: AP, 58.2 mm from the interaural midpoint; ML, 20.3 mm from the intraparietal suture; DV, 17 mm from dura mater. The perfusion rate was 0.2 μl/min for 10 min using an injection microperfusion pump (Mod. 100, Stoelting Co, USA). After Evan´s blue or NaFluo perfusion the needle was rested for 7 min and then withdrawn in steps of 1 mm/min [14]. In some experiments, polystyrene pearls of 2- or 0.2-μm diameter were perfused using the same protocol.

### Light and fluorescence microscopy

After the intracerebroventricular infusion of Evans blue, NaFluo or polystyrene pearls the rats were perfused intracardially with PBS followed by paraformaldehyde 4% and sucrose 3% in PBS. In some experiments, 220 μl of Evan´s blue solution or 1.0% toluidine blue solution were administered through the left heart ventricle at the end of the fixation solution, followed by the same procedure used for the dyes and pearls administered intracerebroventricularly [15]. The dura mater sack that encapsulates the pituitary was removed *in situ*, the gland was washed 3 times with 1 ml PBS and then continued with the histological processes. The dissected pituitary was placed in 20% sucrose in PBS for 8 h at 4°C, embedded in a cryoprotect gel (Freeze Mount, BBC Biochemical, Mount Vernon, WA, USA) and cut into 50-μm thickness slides using a cryostat (Leica, Germany). The tissue slides were observed, and the images were acquired with a Leica TCS SP8 424 laser confocal microscope, equipped with a Leica HCX PL FLUOTAR 10x/0.30 DRY and a Leica HC PL APO CS2 63x/1.40 oil objective (Leica, Jena, Germany). Fluorescence microscopy images of the 2 and 0.2 μm polystyrene pearls were obtained with an Axio Observer Z1 microscope equipped with a Zeiss ACHROPLAN 100x/1.25Oil PH3 objective and filters: 38H (excitation 470/40, emission 525/50) and 49 (excitation 365, emission 445/50) (Zeiss, Jena, Germany). Images were obtained with steps of 0.5 μm and then processed using the Image J free software v1.47 and the plugging 3D (NIH; USA). For the light microscopy observations, rats were perfused with paraformaldehyde 4% plus sucrose 3% in PBS, the brain and pituitary dissected and processed for paraffin embedded-HE stain standard technique. The sections were observed and photographed using an Eclipse 80i microscope and NIS-Elements software (Nikon, Tokyo, Japan).

### Tissue sodium fluorescein measurement

The tissue content of NaFluo was quantified according to Schoch et al. [16] with some modifications. Briefly, the pituitary gland without PI and PN, optic nerves, cerebellar lobule IV-V cortex, and media eminence were obtained after NaFluo intracerebroventricular infusion, placed in 200 μl PBS at pH 7.4 and immediately frozen. Then, the tissues were ground in liquid N_2_, 1.2 ml absolute ethanol was added for protein precipitation, and the samples were centrifuged at 13,000 rpm for 20 min at 4°C. The NaFluo concentration was measured in the supernatant at 520 nm emission with an excitation wavelength of 485 nm in a Spectrofluorometer (Infinite 200Pro, Tecan, Männendorf, Switzerland). The protein precipitate was determined by the Lowry method (BioRad, Watfor, UK). Data are presented as fluorescein arbitrary units per protein mg. After intracerebroventricular infusion of NaFluo the tissues were photographed using a smartphone attached to the eyepiece ocular 10X of a stereomicroscope (SMZ645, Nikon, Tokyo, Japan).

### Statistical analysis

Values of tissue fluorescence intensity are expressed as the mean ± standard error of the mean (S.E.M). Differences between the tissue endogenous fluorescence and the NaFluo in the tissues were analysed by one-way analysis of variance (ANOVA) followed by Holm-Sidak´s multiple comparisons test (GraphPad Prism 7.00, San Diego, CA, USA).

## Results

### The pituitary

**Fig 1:**
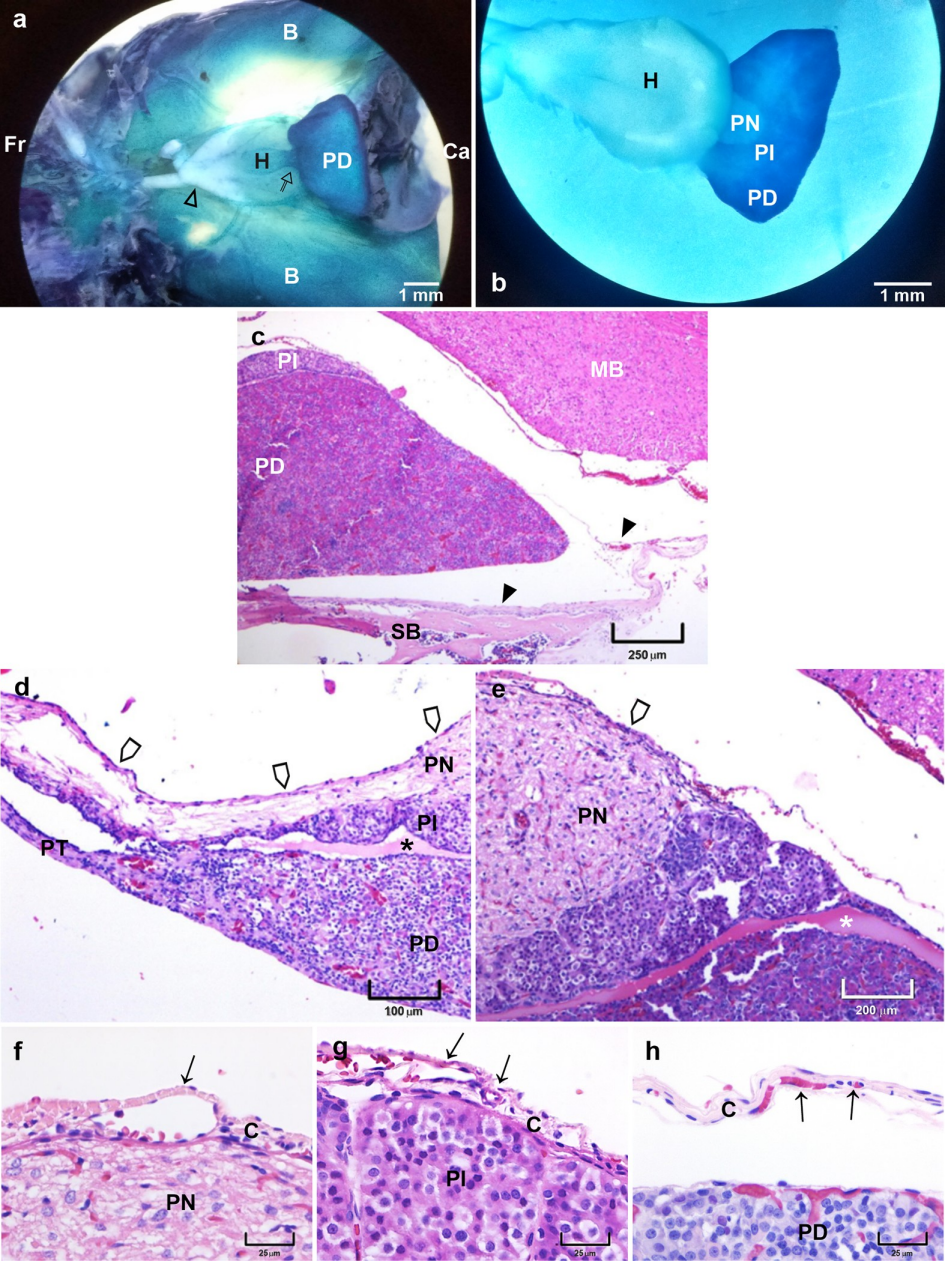
Light microscopy of the pituitary *in situ* and histological sections of the gland. (a) A ventral view of the brain showing position of the pituitary and the different nervous tissues that surround it. (b) Dorsal view of the pituitary and the hypothalamus showing the third brain ventricle. (c) A sagittal view of the pituitary and the adjoining tissues. (d) A middle sagittal section of the pituitary at the most rostral side showing its connection with the hypothalamus. (e) The same pituitary section as (d) but showing the dorsal border where a membrane is observed covering the gland. (f) A detail of the dorsal surface of the PN and the external membrane. (g) A detail of the dorsal surface of the PI showing a space between two lobules and the membrane at the external side. (h) A detail of the border of the PD and the external membrane. Arrow, blood vessel; thin empty arrow, pituitary stalk; empty arrowhead, optic chiasm; arrowhead, dura mater; thick empty arrow, fibrous membrane; asterisk, pituitary cleft. PI, pars intermedia; PN, pars nervosa; PD, pars distalis; PT, pars tuberalis; H, hypothalamus; B, brain; MB, midbrain; SB, sphenoid bone; C, capsule; Fr, frontal; Ca, caudal.

Fig 1 shows different views of the pituitary *in situ*, after the infusion of methylene blue through the cardiac left ventricle observed and sections stained using the HE technique. Fig 1A shows a ventral view of the brain after the sphenoid bone and the dura mater meninges were discarded exposing the pituitary, showing the basal hypothalamus and the connection between the hypothalamus and the pituitary, the pituitary stalk. The pituitary is seen at its ventral side showing only the PD. When the pituitary is isolated with the hypothalamus and showed its dorsal side, it is possible to localize the three lobes of the pituitary (Fig 1B). Fig 1C shows a sagittal view of the brain base where the pituitary is close to the sphenoid bone, and the dura mater meninge covers the gland separated of it. Fig 1D is a rostral mid-sagittal section of the pituitary showing the pituitary stalk and the lobes: PD with the PT, PI and PN, and at the upper side there is a fibrous membrane covering the pituitary stalk and it continues to the PN. Fig 1E corresponds to a caudal section of Fig 1D showing at the dorsal border of the pituitary a membrane covering the gland. Fig 1F-1H show details of the external membrane at the dorsal border and the different lobes of the pituitary. Fig 1F shows the dorsal border of PN and an external fibrous membrane exhibiting a capillary that, at the rightmost, looks separated from the pituitary lobe surface. In Fig 1G is observed a fibrous membrane with multiples capillaries covering two lobules of the PI and at the borders of the lobules is shown a space between them and with the membrane, and in Fig 1H a fibrous membrane with capillaries is shown separated from PD.

### The pituitary capsule

**Fig 2:**
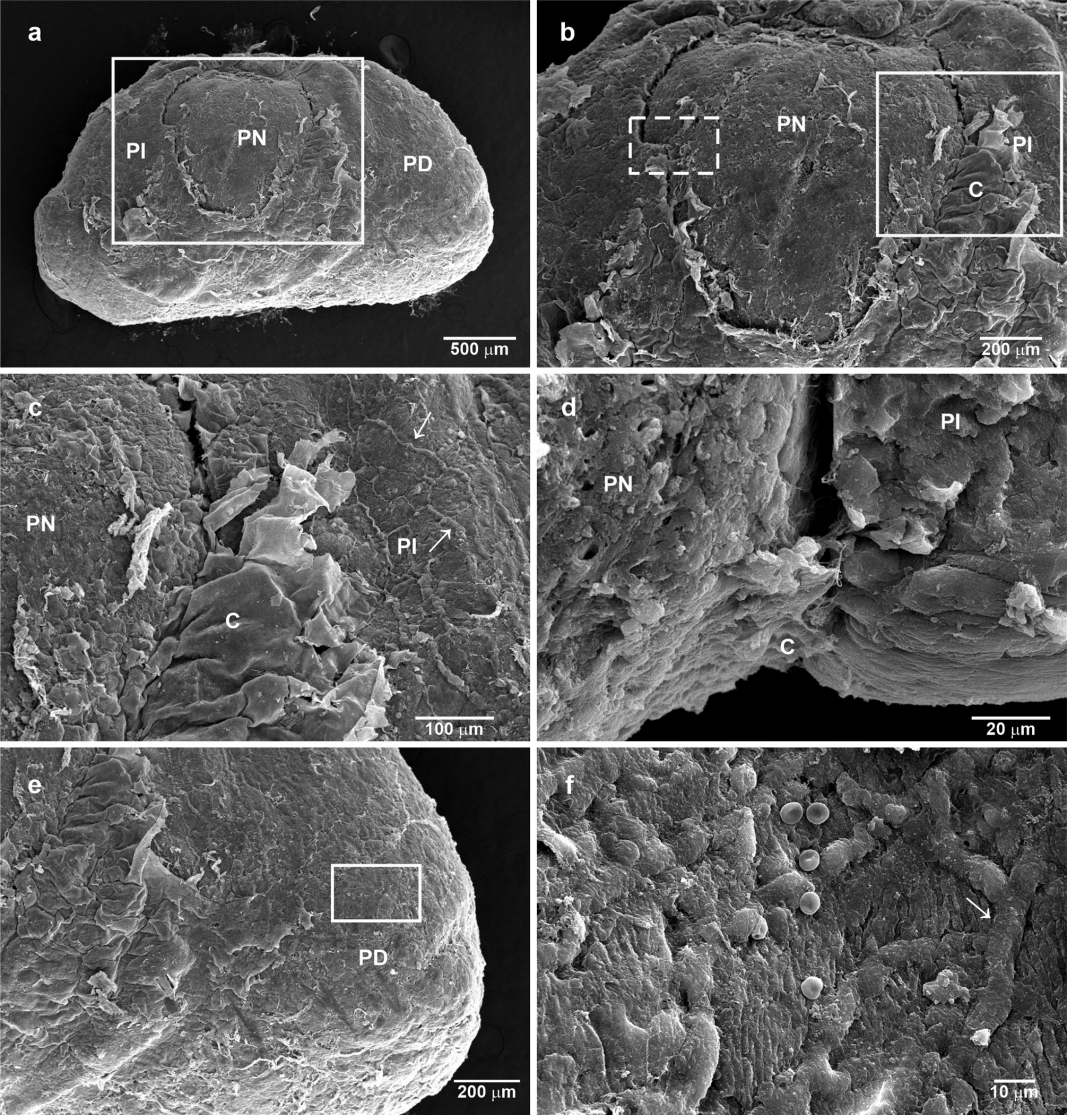
Scanning electron microscopy (SEM) of the exterior of the pituitary gland. (a) A view at low magnification of the posterior face of the pituitary where an external envelope or capsule looks like a thickness layer covering the PI, a furrow delimits the PN and PI borders, and the thicker external envelope becomes thinner at PD. (b) An approach of the zone marked by the square in (a) to illustrate the previous description. The dashed line square is included and described in Fig 2(A). (c) High magnification of the capsule that folds and looks if it surges or invaginates from the border of the PI in the vicinity of PN. (d) A detail of the zone where the PI and PN are close together, showing the furrow between them and the covering or invagination of the thicker capsule of both sides. (e) A view of the PD surface. (f) High magnification of the square in (e), where some erythrocytes are seen on the surface, but more relevant is the presence of abundant blood vessels. Arrow, blood vessels; PI, pars intermedia; PN, pars nervosa; PD, pars distalis; C, capsule.

Fig 2 shows from scanning electron microscopy (SEM) a complete view of the posterior face of the pituitary gland. In this view, it is possible to distinguish the three lobules: PN, PI and PD. The pituitary has a gross external envelope or capsule that covers part of the PN and the PI and extends to the PD (Fig 2A). Fig 2B and 2C shows that the envelope is invaginated at the borders between the PN and PI. In addition, on the PI side, there are many blood vessels. A transversal cut at the middle of the PN exhibits a space between both pituitary lobules where the external envelope comes in (Fig 2D). If one moves towards the PD where the external envelope gets thinner and makes a zoom in this area (boxed area), multiple vessels could be observed (Fig 2E and 2F).

**Fig 3:**
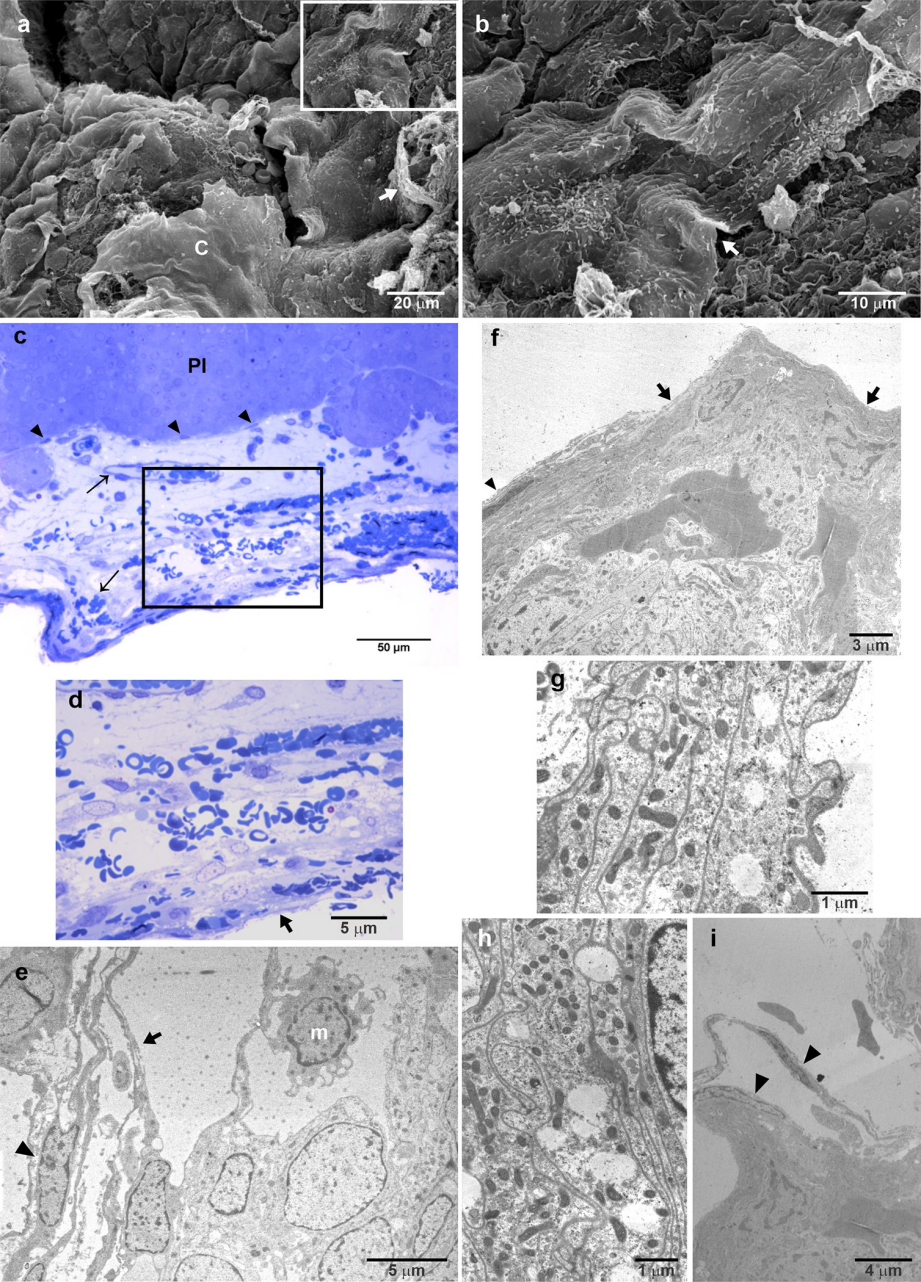
Characteristics of the capsule that covers pars intermedia. (a) SEM of the capsule detail (dashed line square from Fig 2(B) that covers the PI. (b) High magnification of the rectangle in (a) to appreciate the folded and fibrous aspect of the pituitary capsule. Note the digitiform protrusions and the multiple fibrous layers. (b) Higher magnification of the surface of a capsule fold with digitiform protrusions and showing multiple fibrous layers. (c) Semithin sections of the PI covered by the capsule, which is similarly to lose connective tissue. The PI consists of a relatively large amount of amorphous extracellular matrix with capillaries and different types of cells. Moreover, the PI and the capsule are separated by a thin fibrous layer and fibroblast-like cells from the capsule, whose external limit looks denser and thicker, with fusiform cells and peripheral capillaries. Some erythrocytes were observed on the capsule surface. The rectangle is observed as a higher magnification of the capsule in (d). (e) Transmission electron microscopy (TEM) of the loose connective tissue with fibrocytes forming well-defined and clear spaces where macrophages-like cells with cytoplasmic protrusions were frequently observed. Other less differentiated cells with large nuclei and abundant euchromatin were located outside the spaces. (f) TEM of the external limit of the capsule showing lamellar organization of overlapping cytoplasmic prolongations from fibrocytes. Long and flattened fibroblast-like cells were observed at the outermost layer of the capsule. These connective tissue elements surrounded the large diameter and sinuous capillaries. (g) Some places of the outermost lamellae of the capsule had fusiform fibroblast-like cells limiting clear and well-defined spaces. (h and i) High magnifications of overlapping cytoplasmic prolongations from fibrocytes, showing numerous mitochondria and interdigitations. Arrow, capillary; arrowhead, fibroblast-like cell; thick arrow, fibrous layers; PI, pars intermedia; C, capsule.

Fig 3 shows the characteristics of the membrane that covers PI. Fig 3A shows SEM micrographs and corresponds to the punctuated box area in Fig 2B and shows details of the thicker folded external envelope with fibrous borders and a complex surface, as can be observed in Fig 3B. Fig 3C shows a semithin section of the PI and its capsule whose structure resembles leptomeninges. At the border of the PI, a thin layer with fibroblast-like cells separates the glandular tissue from the capsule. It is possible to observe capillary vessels surrounded by pericytes and fibroblast-like cells embedded in loosely arranged connective tissue where cells with a less differentiated morphology are present (Fig 3C). At the external border, the capsule appeared denser and thicker with many erythrocytes among the connective tissue, suggesting a very thin wall of capillaries at this level (Fig 3C and 3D). Additionally, there were long and slender cells with elongated nuclei, such as fibroblast cells, at the innermost aspect of the capsule (Fig 3C). In summary, the semithin section photomicrographs allowed us to distinguish various types of cells according to their size, nucleus shape, and chromatin arrangement (Fig 3C and 3D). Fig 3E shows details at the transmission electron microscopy (TEM) of the cells observed in the loosely arranged connective membrane where at least three different types of cells can be identified: cells with thin membrane protrusions and dentate nucleus and marginated heterochromatin, a macrophage cell; a cell with a larger and paler nucleus than the other cells, a trabecular cell; a fibroblast-like cell with an elongated nucleus surrounded fibrous fibres. The envelope or capsule of the pituitary has an external fibrous layer and fibroblast-like cells densely packed in a fibrous membrane (Fig 3C-3F). Below this fibrous layer, there is the typically complex arachnoid membrane, with intermingled membrane processes rich in mitochondria (Fig 3G and 3H). Details of the fibroblast-like cells at the outermost border of the capsule are shown in Fig 3I.

**Fig 4:**
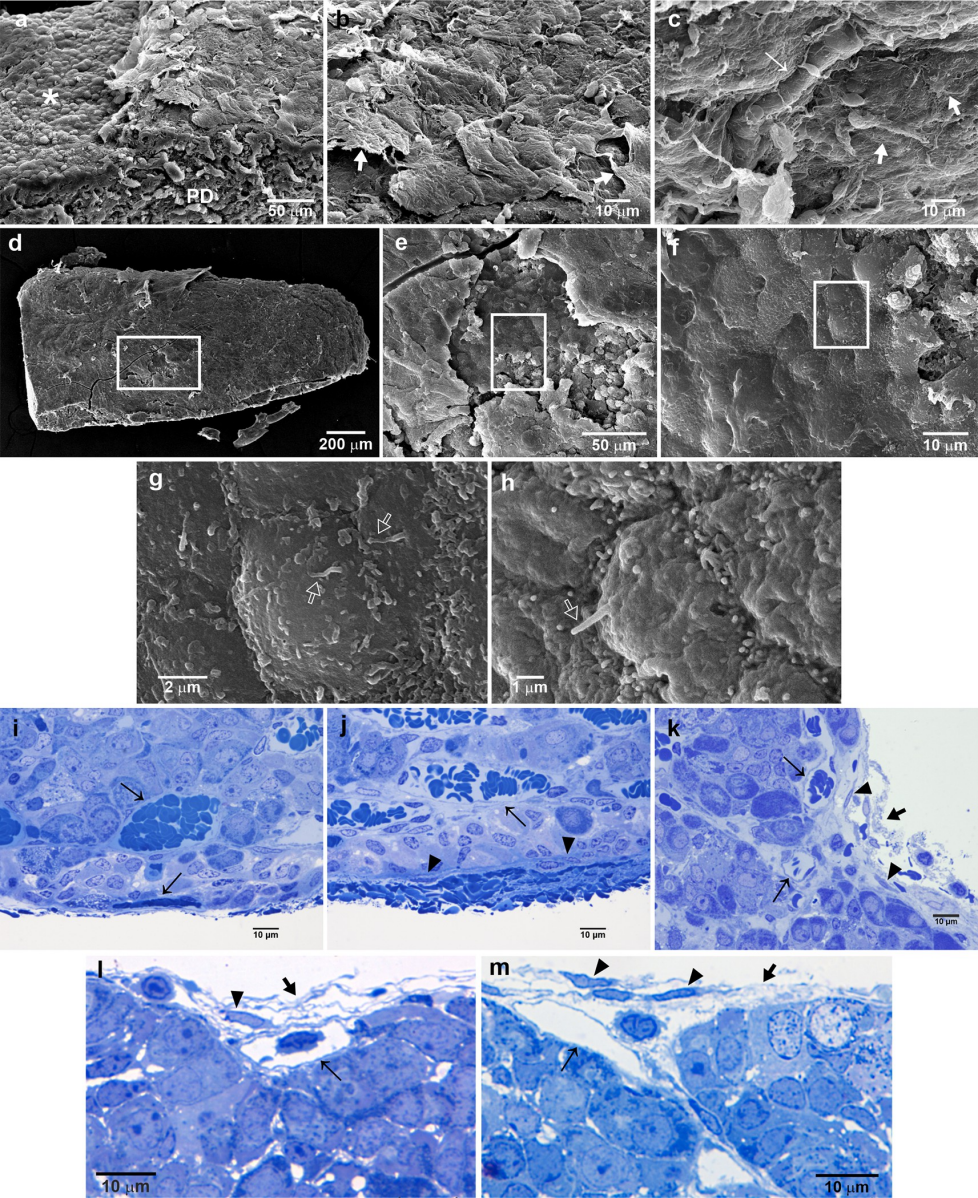
Characteristics of the pituitary capsule covering the dorsal-lateral side of the pituitary gland. (a) SEM of the multiple-layered structure of the capsule is evident, bordering the anterior epithelium of the pituitary cleft and covering the PD. (b) A higher view of the rectangle in (a). (c) SEM of the capsule at the lateral side of the PD showing its multiple-layered, surface cells and a capillary. (d) SEM of the lateral aspect of the PD showing the site where the capsule was torn. (e) A higher magnification of the square in (d) shows the exposure, with numerous cells protruding towards the surface, (f) superficial PD border showing cells with smooth surface some of which show very short primary cilia-like structure, as it is observed at higher magnification in (g and h). (i and j) Semithin sections of the outer surface of the PD showing the multilayered capsule, with embedded capillaries and fibroblast like cells, which is thicker and more complex towards the dorsal lateral aspect of the PD. (k) A view more at the dorsal-lateral aspect than in (j) but also displays organization with invaginations at its more dorsal surface. Note the clear multilayered fine aspect and the space formation in the capsule; these spaces contain lymphocyte- and macrophage-like cells (l and m). Arrow, capillary; thick arrow, fibrous layers; empty arrow, cilium; arrowhead, fibroblast-like cell; asterisk, anterior face of the cleft; PD, pars distalis.

The PD, at the most posterior level, was also seen in the SEM and was covered by a thick multilayer capsule, constituted of multiple fibrous layers with capillaries, and fibroblast-like cells (Fig 4A-4C). When the capsule is broken using the Tanaka´s technique (Fig 4D), it is possible to observe the thickness of the capsule and the surface of the glandular tissue (Fig 4E and 4F). It is interesting that some cells display a short primary cilium-like structure protruding to the space beneath the capsule (Fig 4G and 4H). However, the capsule is not thicker all around the PD, as could be observed in Fig 2 and in the semithin sections in Fig 4I and 4J. A layer of fibroblast-like cells, with long and slender cytoplasmic processes, was in close apposition with glandular cells, as could be observed at the PI capsule (Fig 4K-4M).

**Fig 5:**
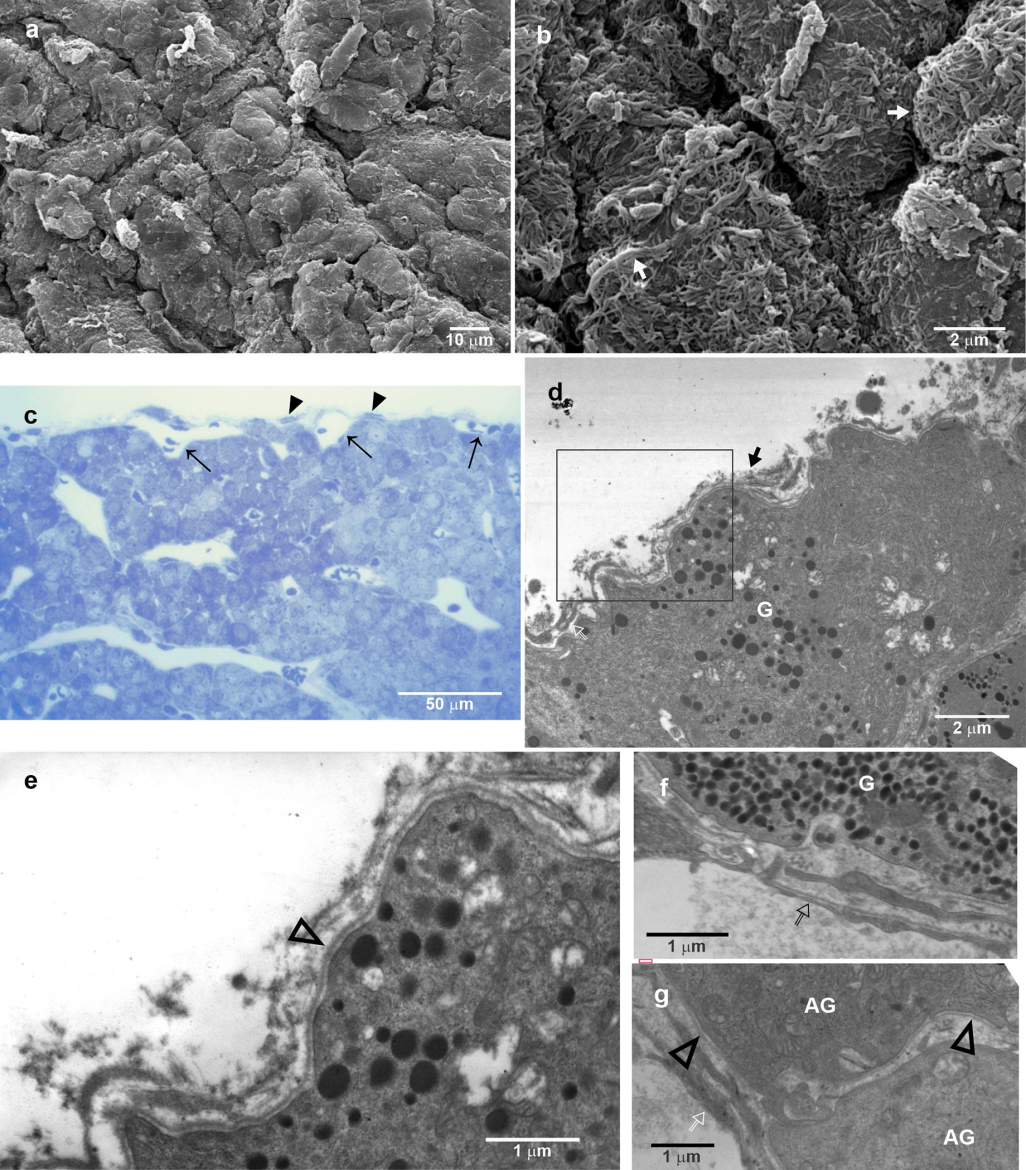
Characteristics of the PD and the pituitary capsule at the ventral side of the gland. (a) SEM of the surface of a PD lateral wing at its more ventral side showing a sinuous and folded appearance. (b) At higher magnification, it is possible to observe the fibrous and reticular material that constitutes the capsule. (c) A semithin section showing the cellular cord arrangement and different sizes of capillaries of the PD, and at the external surface, a thin mesh of fibroblast-like cells and reticular fibres with capillaries embedded in it. (d) TEM of the lateral border of the ventral side of the PD showing an envelope or capsule form by an extracellular matrix with fibrillar components and by a thin continuous amorphous sheet that closely follows the basal contour of endocrine cells. (e) A higher magnification of the square in (d) showing that this sheet is formed by a *lamina densa* separated from the cell surface by a *lamina lucida* or *lamina rara*. (f) A detail of long and slender, parallel, cytoplasmic processes from fibroblast-like cells of the capsule. The *lamina basal* appears to be discontinuous. (g) The capsule, including the *lamina basal*, was also observed covering two agranular cells. Arrow, capillary; arrowhead, fibroblast-like cell; empty arrowhead, lamina basal; empty arrow, cytoplasmic process; G, secretory cell; FS, agranular cell.

An SEM view of the PD at the more ventral and lateral side shows a sinuous surface of the pituitary with lengthen cords, defined by furrows at the outer edges suggesting the external layer invaginates between the tissue cords (Fig 5A). An SEM view of the PD at the more ventral and lateral side shows a sinuous surface of the pituitary with lengthen cords of glandular tissue (Fig 5A). A semithin section shows that the PD cells are arranged mainly in cords between which are large-bore capillaries. At the outermost surface, there is a thin fibrous mesh, like fine reticular fibres with fibroblast-like cells (Fig 5B and 5C). It was also observed that the fibrous membrane protruded to the external space and that it is attached to the superficial tissue (S1 Fig). In addition, peripheral capillaries are frequently observed in this multilayer capsule, and invaginations or septa formation that fuses with the capillary wall are common (Fig 5C). The TEM of a lateral view showed a capsule formed by an extracellular matrix of fibrils and floccular components and cytoplasmic processes of fibroblast-like cells. Additionally, a thin continuous amorphous sheet closely following the basal contour of endocrine cells was identified. This sheet was formed by a *lamina densa* separated from the cell surface of granular and agranular cells by the *lamina lucida* or *lamina rara* (Fig 5D-5G). The membrane at this level viewed by TEM (Fig 5D) showed a fibrous membrane with floccular material and some elongated cellular processes, where granular cells were separated by a basal membrane, as clearly shown in Fig 5E. In Fig 5E-5G, a space between the PD superficial cells and the capsule is observed, and in Fig 5F and 5G, it is clearly shown that the membrane processes of the capsule cells did not contact the glandular tissue, neither granular cells nor agranular cells.

### The epithelial lining of Rathke´s cleft

**Fig 6:**
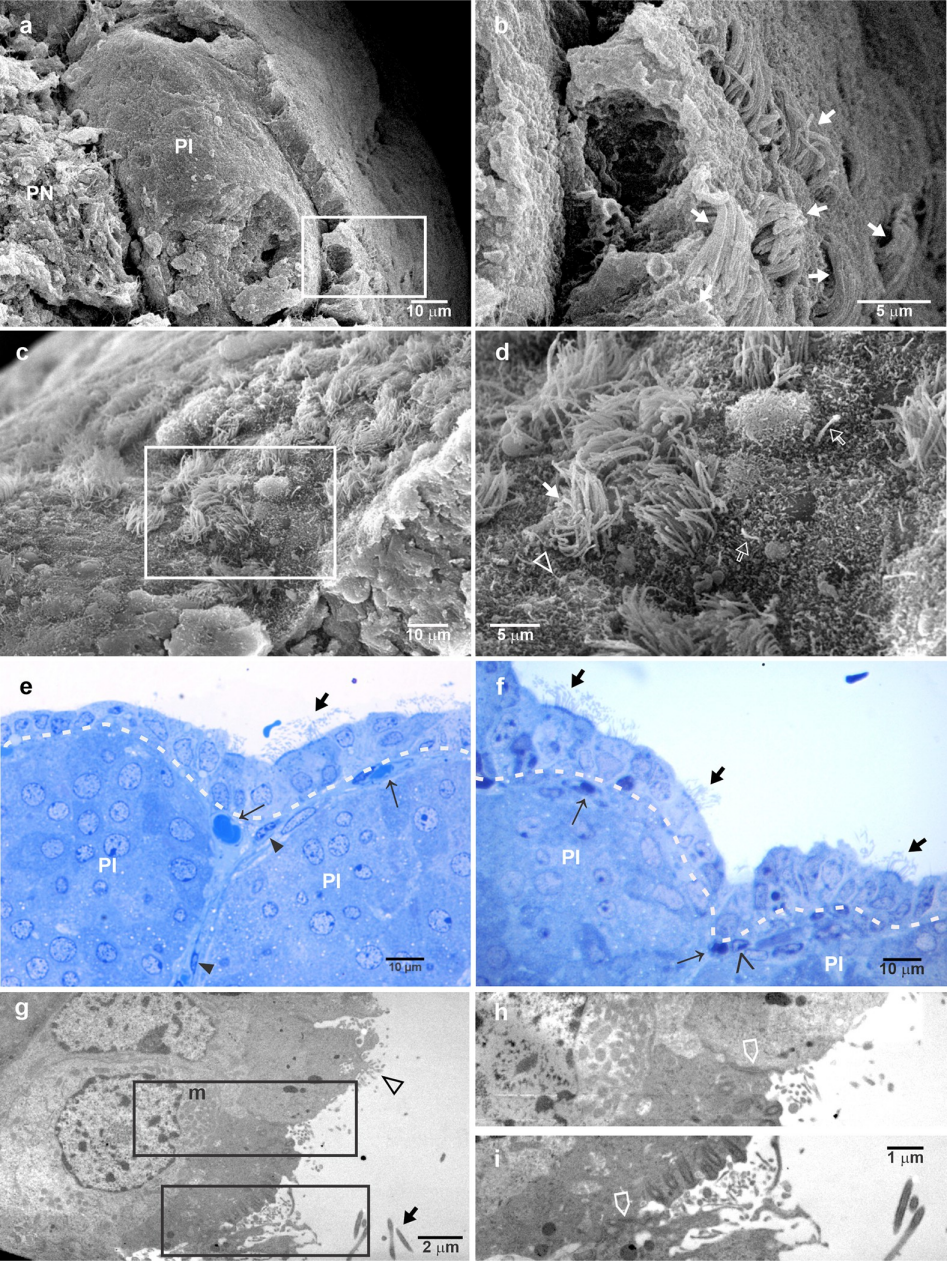
Characteristics of the PI-side epithelial lining of Rathke´s cleft. (a) SEM of the PI and PN and the epithelia of the pituitary cleft as a cuboid layer. (b) Higher magnification of the epithelial layer showing that the main cell type of this epithelium is the multiciliate cell. (c) This epithelium changes at the medial zone, where fewer multiciliate cells are observed. There are cells with microvilli in dome-like apical domain and a third cell type with microfolds and a primary cilium was identified (d). (e and f) Semi thin sections showing the organization of the epithelia as columnar pseudostratified with multiciliate cells and cuboidal cells. The underlying tissue is a scarce *lamina propria* or *stroma* with capillaries, pericytes and fibroblast-like cells, that separates the epithelium and endocrine cells. Connective tissue *septa* from the *stroma* are frequent, dividing the endocrine tissue into lobules. (g) TEM of the posterior epithelia lining the cleft shows columnar pseudostratified organization, where basal mitochondria-rich cells are covered by apical electron-dense multiciliate cells and cells with microvilli. (h) Details of the lateral cell membranes of the apical epithelial cells showing the junctional complexes; no adhesion complexes were observed between the epithelial cells and the cells below them. Arrow, capillary; thick arrow, cilia; empty arrow, cilium; empty arrowhead, microvilli; arrowhead, fibroblast-like cell; empty thick arrow, cell-cell adhesion complex; m, mitochondria; thin arrowhead, pericyte; PI, pars intermedia; PN, pars nervosa.

The PI-side epithelial lining of Rathke´s cleft that covers the PI is formed by a single layer of columnar epithelia in which multiciliate cells predominate (Fig 6A and 6B). However, at the most rostral zone of the PI the epithelial lining changes its cell population. The surface exhibited fewer cilia, and some protuberant apexes with microvilli stood out among ciliated cells (Fig 6C). Areas with a nonciliated or microvilli surface were observed. This zone of the cleft is apparently constituted of epithelial cells whose apical surface carries microfolds, and some present a single or primary cilium (Fig 6C and 6D). Semithin tissue sections showed that the PI-side epithelial lining of the Rathke´s cleft is composed of columnar ciliated and nonciliated cells (with microvilli) and cuboidal cells creating a pseudostratified appearance (Fig 6E and 6F). The epithelium rests on an underlying scarce stroma with capillaries, pericytes and fibroblast-like cells. The epithelium and the melanotrope cells are separated by this stroma or *lamina propria* from which loose connective tissue septa may extend into the interior of PI, dividing it into small lobules or endocrine cell nests (Fig 6E and 6F). TEM corroborates that the posterior epithelia lining the cleft are columnar pseudostratified (Fig 6G). A basal mitochondrial-rich cell type, which apparently does not reach the lumen or epithelial surface, was covered by the electron-dense apical regions of ciliated cells and cells with microvilli (Fig 6G). These cells have round, clear, vacuole-like cytoplasmic spaces or macropinocytic vesicles. Near the apical surface, the lateral cell membranes of these two epithelial cell types form typical epithelial junctional complexes (Fig 6H and 6I).

**Fig 7:**
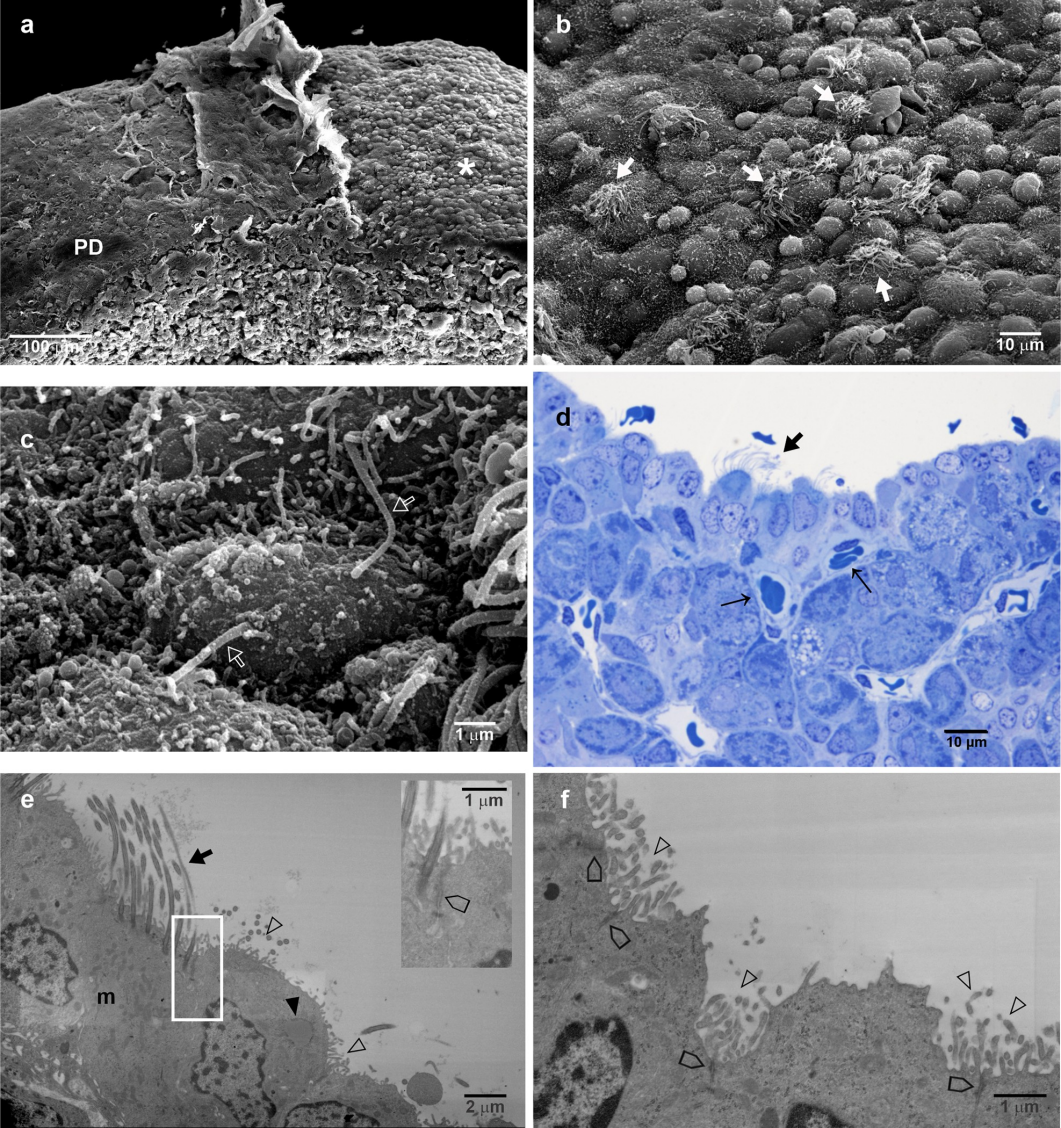
Characteristics of the PD-side epithelial lining of Rathke´s cleft. (a and b) SEM show a surface decorated with apexes of different sizes, some with microvilli irregular in size and shape, other with tufts of cilia, and others rounded and smoother. Flattened areas were also noted at this surface of the Rathke´s cleft. the border of the PD wing and the epithelial cleft layer. (c) Some apexes presented a primary cilium-like structure. (d) A semithin section of the epithelia showing two types of columnar cells, ciliated and with microvilli, whose nuclei are arranged in rows parallel to each other, and some small, superficial cuboidal to flattened cells, with rounded nuclei, are identified. (e) TEM of the epithelial cells where a multiciliate cell, recognized by its basal body, is between two cells with microvilli plentiful mitochondria. The upper right panel shows details of the cell-cell adhesion complexes among epithelial cells. (f) TEM shows cells with irregular shapes and sizes of microvilli, with abundant ribosomes, and some exhibit large droplets of colloidal material. The cell-cell adhesion complexes present in these cells are composed of tight junctions, adherence junctions and desmosomes. Arrow, capillary; thick arrow, cilia; empty arrow, cilium; empty arrowhead, microvilli; arrowhead, colloidal droplet; empty thick arrow, cell-cell adhesion complex; asterisk, anterior face of the cleft; m, mitochondria; PD, pars distalis.

The surface of the PD-side epithelial lining of Rathke´s cleft is characterized by the presence of small, medium, and larger protuberant apexes (Fig 7A-7C), many with microfolds, some with a single or primary cilium (Fig 7C), and few with cilia (Fig 7B). These apexes are interposed among more flattened and smoother surfaces. Semithin tissue sections (Fig 7D) and TEM (Fig 7E and 7F) showed that apexes of the luminal extreme of a lining simple epithelium of pseudostratified appearance by alignment at different levels of nuclei. Three types of cells are recognizable: two types of columnar cells, ciliated and with microvilli, whose nuclei are arranged in rows parallel to each other, and one small, superficial cuboidal to flattened cell, with rounded nuclei. Ciliated cells are readily identifiable by their electron-dense bodies, although they also have numerous irregular microvilli between the cilia (Fig 7E and insert). In addition, cells with only microvilli of irregular size and shape are clearly distinguishable from flattened and ciliated cells (Fig 7E and 7F). Typical epithelial junctional complexes were observed among epithelial cells. Interestingly, ciliated cells have abundant mitochondria, cells with microvilli have abundant ribosomes and some have large droplets of colloidal material (Fig 6E).

### The CSF, PI and PD

**Fig 8:**
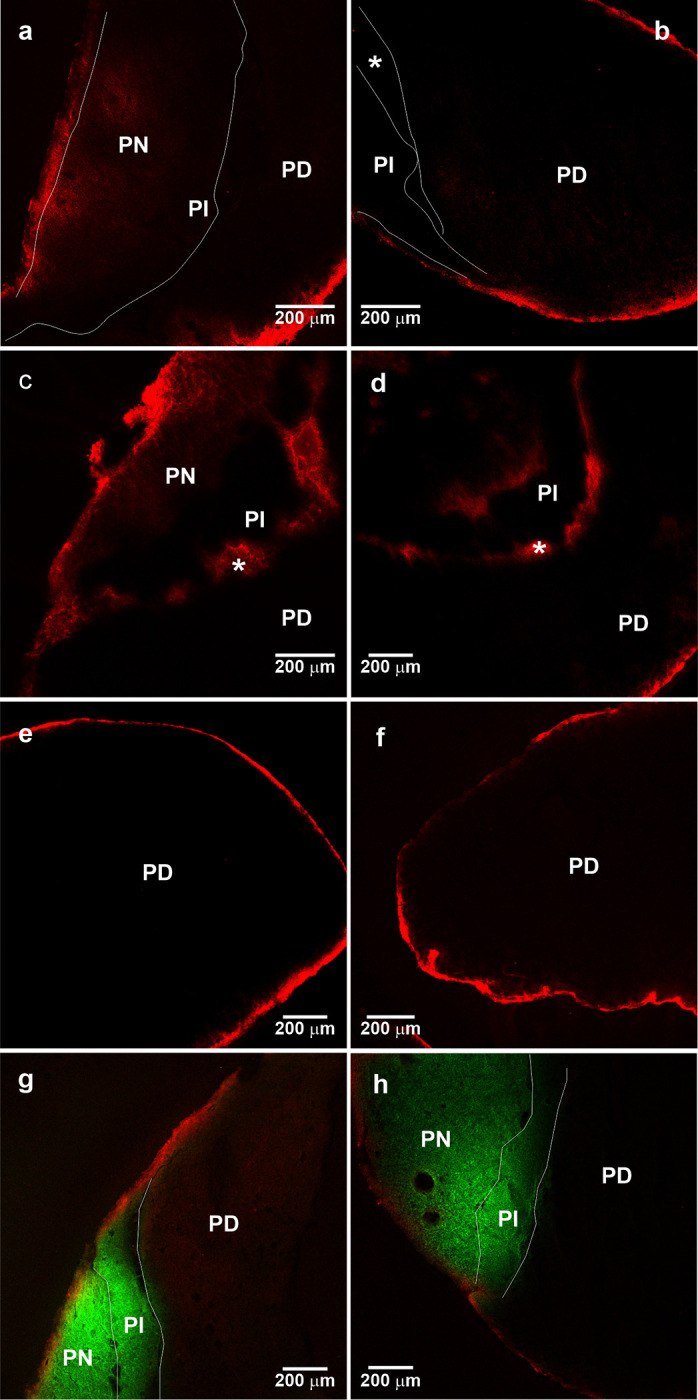
Localization of red fluorescent Evans blue in the pituitary after intracardiac or intracerebroventricular administration of the dye, and of NaFluo administered via intracerebroventricular. (a) Presence of Evans blue at the periphery of the PN and (b) at the periphery of the PD, after the cardiac administration of Evans blue, and the rat nose and ears were bluish. (c and d) Ten minutes later after the intracerebroventricular administration of Evans blue, the dye is observed at the PN meninges, the pituitary cleft, and the PD periphery. (e and f) Two views of the PD surrounded by Evans blue dye intracerebroventricularly administered. (g and h) Two views of the accumulation of NaFluo 40 minutes later after it´s intracerebroventricular administration and followed by Evans blue administered via intracardiac. Evans blue stains in red (excitation = 561 nm, emission = 576/651 nm) and NaFluo in green (excitation = 488 nm, emission = 510/548 nm). Z-projection: (a) 130 μm, (b) 80 μm, (c) 100 μm, (d) 120 μm, (e) 120 μm, (f) 120 μm, (g) 70 μm, (h) 50 μm. PN, pars nervosa; PI, pars intermedia; PD, pars distalis; asterisk, pituitary cleft.

The red fluorescent emission of Evans blue dye was observed at the periphery of the rat pituitary, around PN and PD 10 minutes after its administration through the left heart ventricle (Fig 8A and 8B). It was observed at the periphery of all around the pituitary gland, PN and PD (Fig 8A and 8B) where continuous non-fenestrated capillaries were observed (S2a and S2b Fig). No red fluorescence was noticed in the endocrine and nervous components of the hypophysis, because Evans blue dye was washed with the procedure used to cryo-protect the gland due to the presence of fenestrated capillaries in the pituitary (S2c and S2d Fig); when this procedure was not used, the red fluorescent dye was observed inside the pituitary (S2e Fig). However, 20 min after finishing the intracerebroventricular administration of Evans blue dye, it was observed inside the pituitary cleft, suggesting that the CSF penetrates this space (Fig 8C and 8D). Moreover, Evans blue remained around the whole pituitary, even after the incubation of the gland in sucrose solution (Fig 8E and 8F). Moreover, after 40 min finishing the injection of NaFluo through the lateral brain ventricle, followed by the administration of Evans blue dye through the left heart ventricle, it was observed an accumulation of NaFluo in the PN and PI, excluding PD. This result suggests an infiltration and accumulation of the fluorescent dye dissolved in the CSF, and the Evans blue was observed at the periphery of the whole pituitary (Fig 8G and 8H).

**Fig 9:**
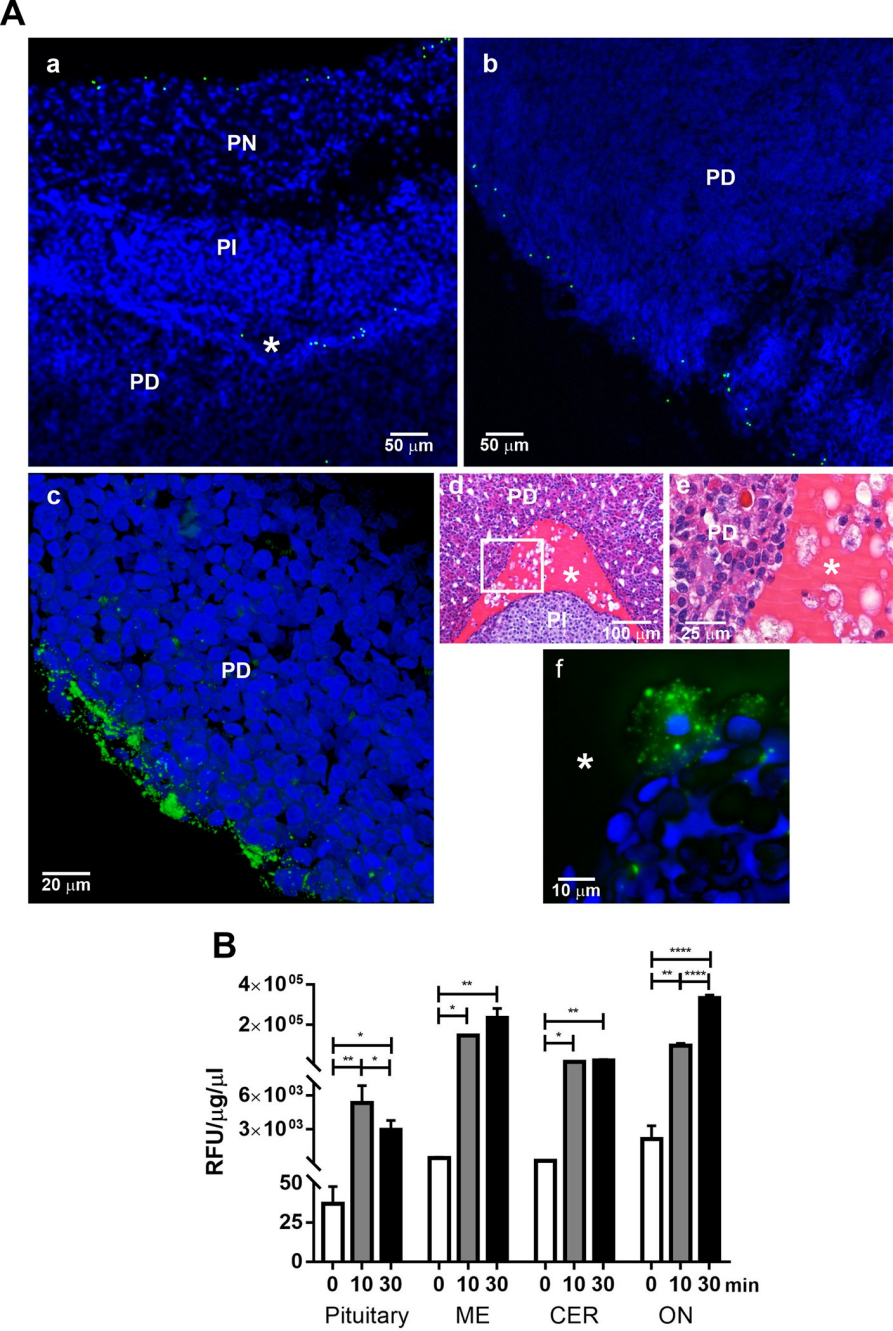
Presence of fluorescent beads and NaFluo accumulation in the pituitary gland after their intracerebroventricular administration. (A) Localization of 2- and 0.2-μm diameter fluorescent beads in the pituitary gland after intracerebroventricular administration. (a) A confocal view of the pituitary where 2-μm diameter fluorescent beads are observed at the PN periphery and inside the pituitary cleft. (b) A confocal view of the PD periphery showing 2-μm diameter fluorescent beads. (c) A confocal view of the periphery of the PD showing an accumulation of 2- and 0.2-μm diameter fluorescent beads. The polystyrene beads are green (excitation = 488 nm, emission = 510/548 nm), the cell nucleus in blue (excitation = 405 nm, emission = 413/479 nm). Z-projection: (a) 94 μm, (b) 36 μm, (c) 27 μm.(d) A view of a pituitary sagittal section stained with HE shows cells inside Rathke´s cleft. (e) Higher magnification of the cells inside Rathke´s cleft. (f) Fluorescens microscopy of a cell at the pituitary cleft that phagocytosed 2- and 0.2-μm diameter fluorescent beads, 20 μm Z-projection. PN, pars nervosa; PI, pars intermedia; PD, pars distalis; asterisk, pituitary cleft. (B) Accumulation of NaFluo after 10- and 20-min of intracerebroventricular administration in different intracranial tissues: Pituitary; ME, media eminence; CE, cerebellar cortex; ON, optic nerve. Differences in fluorescence between the tissue were analyzed by one-way analysis of variance (ANOVA) followed by Holm-Sidak´s multiple comparisons test. Differences between endogenous and NaFluo accumulation MR and AR groups, * *P* <0.05, ** *P* <0.01, ** *P* <0.0001.

Regarding the injection of the 2-μm fluorescent beads, these were observed at the PN edge and in the Rathke´s cleft (Fig 9Aa) and at the PD border (Fig 9Ab). When 0.2-μm fluorescent beads were injected, they accumulated at the posterolateral side of the PD (Fig 9Ac), but they were seen only on the periphery as observed in the 3D image video (S3 Fig). Within the Rathke´s cleft there are cells suspended in colloid (Fig 9Ad and 9Ae). When 2- and 0.2 μm fluorescent beads were injected intracerebroventricular, they entered the cleft and were phagocytosed by cells inside the cleft space (Fig 9Ac, and S4 Fig). After the administration of NaFluo through the left lateral brain ventricle and at different time intervals, various intracranial tissues were obtained, and their NaFluo accumulation was measured: pituitary, media eminence (EM), cerebellum cortex (CER) and optical nerve (ON). From the pituitary, only the PD was analysed. It is important to emphasize that the samples were washed three times with PBS after they were processed. The initial fluorescence level was the endogenous fluorescence. Following intracerebroventricular NaFluo administration, a statistically significant increase in fluorescence emission in all the samples was observed from 10 min after the infusion until 30 min later (Fig 9B). This increment was 113 and 63 times more in the PD, 319 and 512 times more in the EM, 65 and 98 times more in the CER, and 45 and 154.5 times more in the ON at 10 and 30 min, respectively. The accumulation of fluorescein after 10 min of NaFluo administration was easily observable macroscopically and showed clear differences between the samples studied (S5 Fig).

## Discussion

Our results showed that the rat hypophysis capsule has different thicknesses around the gland. At the posterior-coronal side, the capsule is structured by a membrane with different layers of fibroblast-like cells, a loose mesenchymal membrane with cells with large pale nucleus, followed by an intricate layer of membrane processes rich in capillary vessels and at the more external surface a fibrous layer, suggesting that at this level the capsule corresponds to a leptomeninges organization [17, 18]. Furthermore, Krish and Buchheim [4] described the rat capsule as a meningeal membrane. However, unlike brain leptomeninges in mice [17], the pituitary has an external fibrous layer and fibroblast-like cells densely packed in a fibrous membrane. The human pituitary envelope has been studied more extensively [19], and it has been described to consist of two membrane layers: a lamina propria covering the external surface of the gland, and an outer fibrous layer, the capsule, with a space between them [3]. Moreover, the capsule is thicker at the inferolateral region than the other parts of the gland [3]. SEM showed that the capsule is thick at the PN and the PI but is not a continuous layer because the membrane passes through a space between both lobes. A micrograph obtained by Wada et al. [20] shows a complex organization of the capsule at this level. However, the capsule at the PI continues towards the PD, and although it is less thick, it is structured by different layers of fibroblast-like cells, and a dense fibrous layer with cell characteristics similarly to those present at the thick capsule of the PI. Then, the gross capsule that resembles the leptomeninges also covers part of the PD; however, at the more anterior-rostral side of the gland, there seems to be a thin layer of fibroblast-like cells immersed in a thin fibrous membrane. According to Mesey and Miklós [1], only PN and PT are covered by leptomeninges, but Guerra et al. [8] showed that at PT the arachnoid is loose, leaving only the pia mater membrane covering this part of the pituitary. Our observations suggest that the pia mater continues to the PD, in agreement with the observations of Guerra et al. [8] in the PT and the conclusion of Ciric [2] in human pituitaries. The relationship between the capsule and the pituitary parenchyma shows that they are separated by a basal membrane or lamina propria covering the pituitary surface, as can be observed in this study and what was reported by Krish and Buchheim [4]. Moreover, we observed that the membrane covering the surface is attached to the surface of the superficial tissue, as was reported by Krisch and Buchheim [4]. Nevertheless, Schechter [21] observed that there are discrete zones at the parenchymal border where gonadotrophs protrude through the basal membrane and could release factors to the meningeal capsule, such as fibroblast growth factor. The basal membrane and the capsule are separated, and CSF fills the space, in accordance with our observations with the presence of Evans blue and the polystyrene pearls at the borders of the gland, and with the measures of NaFluo accumulation at the surface of the PD. We also observed the presence of cells where a cilium protrudes towards this space, suggesting that they can receive signals from the CSF. According to the volume transmission communication concept, the pituitary could receive messages from different brain areas through the CSF and, as Veening and Barendregt [12] postulate, it could respond in accordance with the different brain states. Jindatip et al. [22] described a novel pituitary cell that is desmin-positive and exhibits a cilium; however, they reported that this cell is localized in the capillary vicinity. Not only is the surface of the pituitary bathed with CSF, but we could also localize the Evans blue dye and polystyrene beads inside the pituitary cleft and NaFluo accumulated in PN and PI after they were infused through the lateral ventricle of the brain. It was interesting to find the presence of the 2-μm diameter fluorescent pearls in this space. The entrance of the CSF to the pituitary cleft could be explained if it is considered a continuation of the PT cisterns to the cleft [8, 23]. Another characteristic present in the overall capsule is the presence of capillaries, which are abundant and correspond to the capsular network [24]. According to Murakami et al. [24], the capillary capsular network receives its arterial twigs mainly from the internal carotid and posterior hypophyseal arteries and is connected into the neurohypophyseal, dorsal adenohypophyseal and ventral adenohypophyseal veins. According to our observations when Evans blue was administered intracardially, this capillary network is vessels that are continuous non-fenestrated, as Krish and Buchheim [4] reported and we also observed. Considering that these vessels are in a meningeal context, it is interesting to note that they are capillaries, differing from the rest of the pial vasculature which are arterioles and venules [25], suggesting that they can regulate the free movement of molecules. Further studies are necessary to characterize the function of these capillaries.

When we looked for the cell organization of the epithelia covering the pituitary cleft, also named the Marginal Cell Layer (MCL), and in accordance with Ciocca [10] and Correr and Mota [11], we observed an abundance of ciliated cells at the posterior face localized forward to PI, and the PD-side exhibits an epithelium with fewer multiciliate cells. However, at both epithelial layers there are other cells with multiple microvilli and others presenting a cilium at their apical surface. It was interesting to find that cells below the epithelia are clustered with the multiciliate and microvilli cells forming a unit. In accordance with Ishii and Ishibashi [26], we observed that no adhesion complexes are present between the epithelial and the cells below them. Moreover, they are separated from endocrine cells by a connective thin membrane. These units are more defined at the PI-side epithelial lining of the Rathke´s cleft, but at the PD-side, these units are rich in capillaries. The observations at the TEM showed epithelial cells at the surface jointed with the typical epithelial adherence complexes: zonulae occlude, zonulae adhere and desmosomes, and the cells below them have abundant mitochondria. Together with this epithelium are cells that present a cilium, as seen in the rest of the superficial layer of the PD. The data suggest that the epithelia lining the surface of the cleft moves an external liquid medium in addition to sensing its composition. Supporting this suggestion is our observation of dyes dissolved in the CSF inside the pituitary cleft. Moreover, the epithelia of both layers of the cleft express aquaporin 4, and specifically, the multiciliate cells express aquaporin 5 [27]. Moreover, it has been reported that multiciliate epithelial cells of the MCL express the transcription factor FOXJ1, an essential expression factor for ciliogenesis, and together with the transcription factor SOX2 maintains the phenotype of these cells [28]. The expression of aquaporin 4 in the brain is well known to be involved in fluid transport into and out of it; therefore, it is an important participant in the volume control of the brain [29]. However, a recent study by Horiguchi et al. [30] showed another function of ciliated cells: they express the enzymes that synthesize and degrade retinoic acid and they suggested that as they adjust the retinoic acid concentration, they regulate the stem cell niche of the pituitary cleft. At the border of the pituitary cleft, constituted by the MCL, it is well known that there are SOX2+ progenitor cells [31].

It has been established that the principal regulator of the pituitary is the hypothalamus, which releases factors to the portal vessels, where they reach the pituitary cells. These factors are messengers of the integratory response of the nervous system and control the secretory function of the pituitary cells. Other inputs for the pituitary arrived by circulating blood. The efficient response of the pituitary is ensured by its tissue organization in homotypic and heterotypic networks [32, 33]. In the PT, Guerra et al [8] observed that the CSF bathes its interior by a cistern system. This observation is important because PT is recognized as a structure for the regulation of seasonal rhythms for reproduction and is related to metabolic and immune functions, which respond to melatonin and its photoperiod and temperature variations through thyroid stimulating hormone (TSH) [34, 35]. Melatonin reaches its target tissues in two ways, the bloodstream and CSF, and through the latter, a strong stimulus is achieved [36]. Our observations show CSF bathing the pituitary cleft, suggesting that this communication path could be used by the PI and PD with the PT and median eminence system. Moreover, Horiguchi et al. [30] showed that epithelial cells of the posterior layer synthesize retinoic acid, and melatonin is known to stimulate its synthesis in tanycytes participating in the circannual regulation of body weight and breeding [37]. Moreover, we hypothesized that signals from the CNS can reach the secretory parenchymal cells of the PD travelling in the CSF and be recognized by the ciliated cells present on the surface of the PD and the epithelium of the pituitary cleft. Recognized pituitary cells exhibiting a single cilium are localized: at the PT and are secretory cells [8], at the pituitary cleft [10], at the PD in the parenchymal follicles and are follicle-stellate cells [38], and at the blood vessel periphery and are desmin-immunopositive cells [22]. We now added a non-identified cell at the surface of the PD and facing the pituitary capsule. What is the physiological function of these peripheral cells and how do they transmit what they perceive? According to the volume transmission communication, through the CSF neuroactive substances travel nearby or long distances, reaching the ventricular epithelia and outer surface of the brain and inducing the secretion of neuromodulators which contribute to regulate brain states [12, 13]. The cellular function of the ciliate peripheral cells and those of the Rathke´s cleft epithelia we have described could be the transmission pathway of CSF signals to the pituitary coordinating it with other brain areas. However, further studies must be performed. Moreover, it is recognized at the PD an intricate network of follicle-stellate cells, which are characterized as a heterogeneous population with different electrical responses and present gap junctions allowing a large-scale intrapituitary communication [39]. Interestingly, Sato et al. [40] described that follicle-stellate cells are abundant at the basal periphery zone and at the cranial and caudal regions. According to our observations, at the caudal region, the capsule is thick and rich in capillaries, suggesting a zone of interchange between blood and CSF. Our study showed that the CSF bathes the pituitary gland suggesting that there is another way to intercommunicate the CNS and the gland through the CSF.

## Supporting information

S1 Fig SEM of the capsule that covers the surface of the PD. (a) A lateral view of the PD wing showing part of a membrane with a soft surface. (b) A high magnification of the membrane. (c) A fibrous layer surging from the surface covering the PD. (d) A higher magnification of (c) showing that it is constituted of fibrous strands. PD, pars distalis.
(TIF)

S2 Fig TEM of capillaries of the capsule and the PD tissue, and a confocal image of PD capillaries after an infusion of Evans blue dye through the left cardiac ventricle. (a) A capsule capillary, (b) a higher magnification of (a) of a zone of endothelial multiple adhesion complexes, (c) a PD capillary, (d) a higher magnification of (c) showing fenestrations, (e) a confocal micrograph of PD capillaries filled with Evans blue dye in red (excitation = 561 nm, emission = 576/651 nm; Z-projection 40 μm). Arrows, blood vessel; thick arrow, adhesion complex; empty thick arrow, fenestra.
(TIF)

S3 Fig Accumulation of fluorescent polystyrene beads at the PD edge after their intracerebroventricular injection. 3D image of the periphery of the PD showing the accumulation of 0.2 μm diameterfluorescent polystyrene pearls. PD, pars distalis.
(AVI)

S4 Fig A view of a cell at the Rathke´s cleft that phagocytes polystyrene beads after their intracerebroventricular injection. 3D image of a cell inside the cleft space that have engulfed 2- and 0.2 μm diameter fluorescent polystyrene pearls.
(AVI)

S5 Fig Observation of tissues isolated after the intracerebroventricular administration of NaFluo. (a) P, pituitary. (b) CER, cerebellum cortex. (c) ON, optic nerve. (d) ME, median eminence. Key words: pituitary capsule, cerebrospinal fluid, leptomeninges, Rathke´s cleft epithelia.
(TIF)
