# Supplementary figures and images for: Characterization of the rat pituitary capsule: Evidence that the cerebrospinal fluid filled the pituitary cleft and the inner side of the capsule
Source: PLoS One. 2023 May 26;18(5):e0286399. doi: 10.1371/journal.pone.0286399 (PMC10218750; doi:10.1371/journal.pone.0286399)

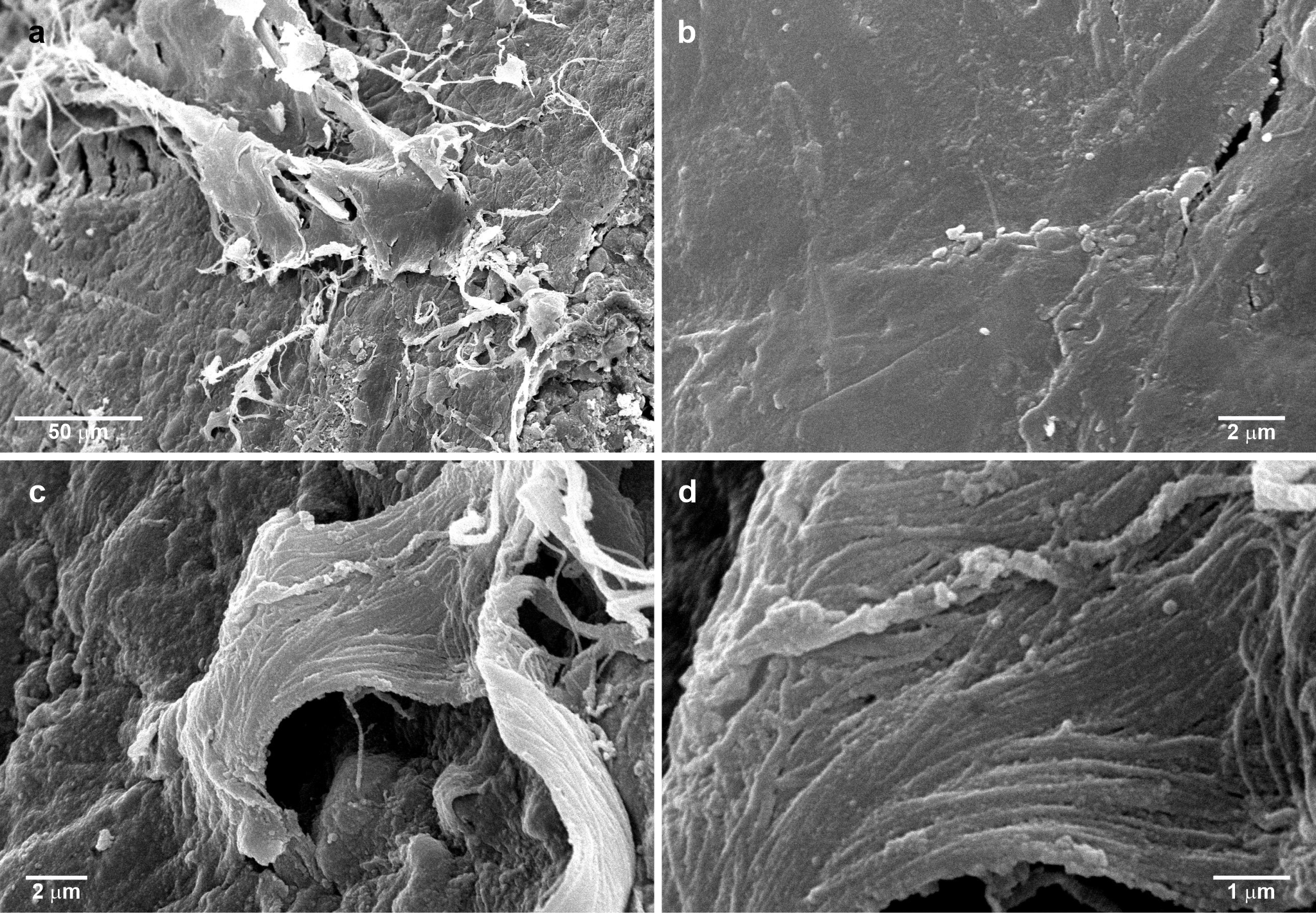

Supplement: S1 Fig — (a) A lateral view of the PD wing showing part of a membrane with a soft surface. (b) A high magnification of the membrane. (c) A fibrous layer surging from the surface covering the PD. (d) A higher magnification of (c) showing that it is constituted of fibrous strands. PD, pars distalis. (TIF) [file pone.0286399.s001.tif]

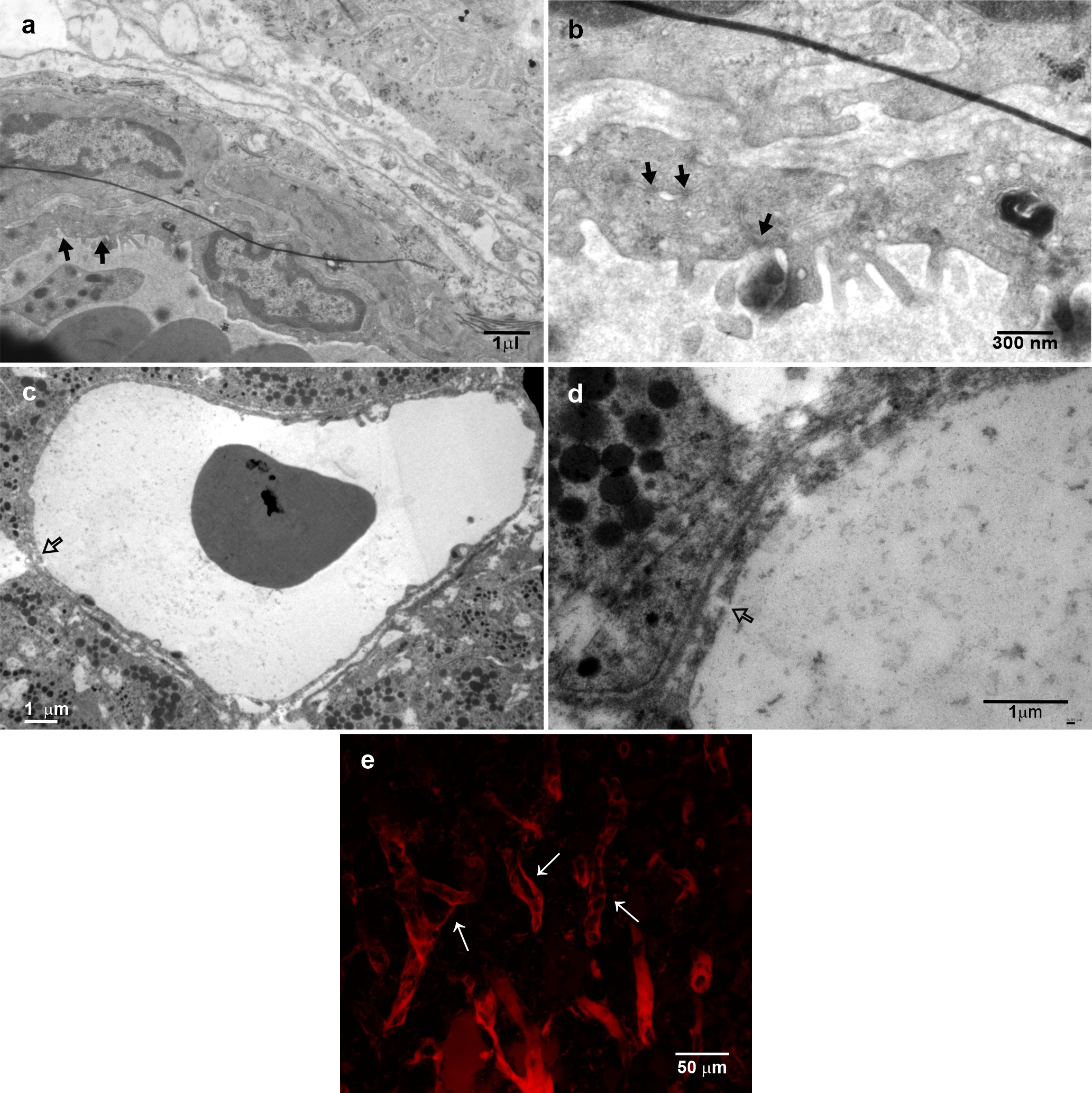

Supplement: S2 Fig — (a) A capsule capillary, (b) a higher magnification of (a) of a zone of endothelial multiple adhesion complexes, (c) a PD capillary, (d) a higher magnification of (c) showing fenestrations, (e) a confocal micrograph of PD capillaries filled with Evans blue dye in red (excitation = 561 nm, emission = 576/651 nm; Z-projection 40 μm). Arrows, blood vessel; thick arrow, adhesion complex; empty thick arrow, fenestra. (TIF) [file pone.0286399.s002.tif]

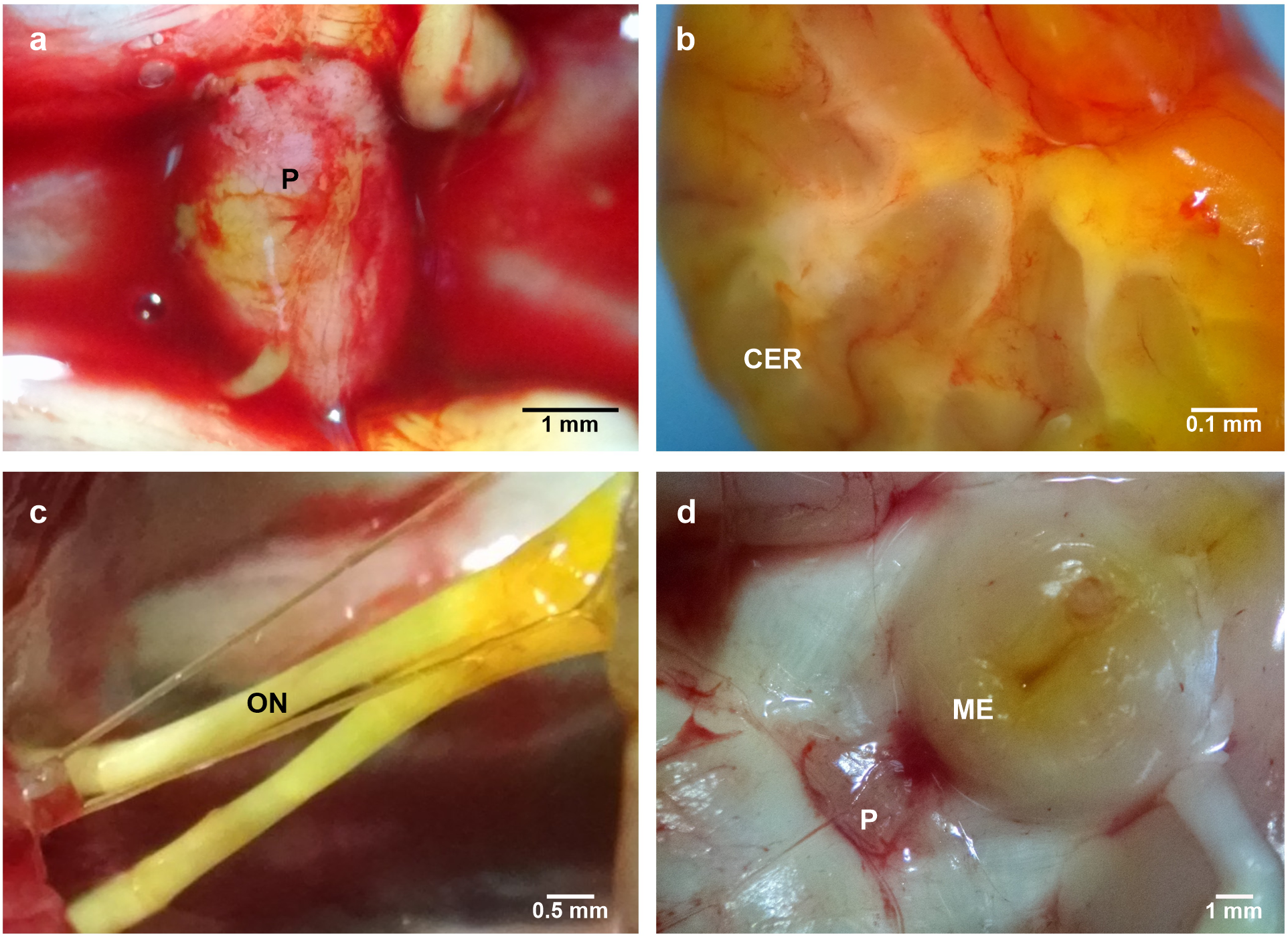

Supplement: S5 Fig — (a) P, pituitary. (b) CER, cerebellum cortex. (c) ON, optic nerve. (d) ME, median eminence. Key words: pituitary capsule, cerebrospinal fluid, leptomeninges, Rathke´s cleft epithelia. (TIF) [file pone.0286399.s005.tif]
